# Morphological changes in smectic liquid crystal microstructures

**DOI:** 10.1039/d6sm00042h

**Published:** 2026-03-17

**Authors:** Daichi Sato, Yutaka Sumino, Takahiro Yamamoto, Igor Muševič, Yoshiko Takenaka

**Affiliations:** a Department of Applied Physics, Faculty of Advanced Engineering, Tokyo University of Science 6-3-1, Nijuku Katsusika-ku Tokyo 125-8585 Japan; b Research Institute for Sustainable Chemistry, National Institute of Advanced Industrial Science and Technology 1-1-1, Higashi Tsukuba Ibaraki 305-8565 Japan takenaka.yoshiko@aist.go.jp; c Water Frontier Research Center and Division of Colloid Interface, Research Institute for Science & Technology, Tokyo University of Science 6-3-1, Nijuku Katsusika-ku Tokyo 125-8585 Japan; d Faculty of Engineering and Physical Sciences, University of Surrey Guildford Surrey GU2 7XH UK; e Department of Condensed Matter Physics, Jožef Stefan Institute Jamova cesta 39 SI-1000 Ljubljana Slovenia; f Physics Department, Faculty of Mathematics and Physics, University of Ljubljana Jadranska 19 SI-1000 Ljubljana Slovenia

## Abstract

We report temperature-induced morphological transitions of smectic liquid crystal (LC) microstructures from fibers to disc- and umbrella-like structures. We used two systems based on 4-cyano-4′-*n*-octyloxybiphenyl (8OCB): an 8OCB/decanol system and an 8OCB/cetyltrimethylammonium bromide (CTAB) system. In both systems, LC fiber structures grew from the droplets upon cooling. The LC fiber structures in both systems underwent similar morphological transitions into umbrella-like structures *via* an intermediate disc-like structure. Furthermore, we observed that repeated temperature cycling induced reversible morphological transitions between umbrella- and disc-like structures. We developed a simple free-energy model, based on elastic and topological defect energies, that explains these morphological changes. These findings suggest design principles for stimuli-responsive smectic LC microstructures and may provide physical insight into the deformation of phase-separated, membraneless organelles.

## Abstract 2

Temperature-driven deformation of smectic liquid crystal microstructures reveals reversible transitions between umbrella-like and disc-like structures.

## Graphical abstract

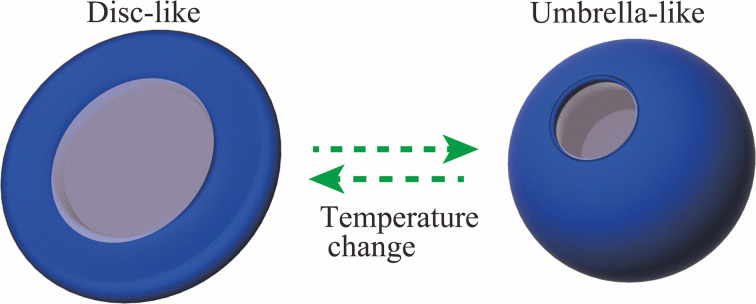

## Introduction

It is important to clarify and understand the nature of common mechanisms that induce three-dimensional geometry, *i.e.* shape changes in various condensed matter systems such as vesicles, biomembranes, and liquid crystals (LCs).^1,2^ Of particular importance are systems that can spontaneously change their shape in response to specific external stimuli, providing a variety of means for transporting materials and controlling their physical properties. In recent years, research has been actively conducted on the morphological changes of microstructures using LC molecules.^2–5^

Liquid-crystalline systems are broadly divided into lyotropic liquid crystals (LyLCs) and thermotropic liquid crystals (ThLCs). In LyLCs, the phase behavior depends sensitively on both concentration and temperature,^6^ whereas in ThLCs, it is typically discussed in terms of temperature-dependent transitions at the fixed composition. The biological relevance of LyLCs, particularly phospholipids, has stimulated extensive research and led to a substantial accumulation of knowledge.^7–9^ Such fundamental studies have enabled the application of LyLCs under both *in vivo* and *in vitro* conditions. In the context of drug delivery, for example, LyLC-based vesicles have been utilized as soft carriers capable of deformation and even self-propulsion. Vesicles formed from LyLCs exhibit a variety of dynamic behaviors, such as environmentally responsive shape changes and autonomous motion, which have led to their exploration as candidates for drug carriers and micrometer-scale biomimetic models.^10–14^ Previous studies have shown that vesicles undergo a range of deformation processes, including transitions to discocyte (defined here as a disc-like structure) and stomatocyte (defined here as an umbrella-like structure) morphologies.^15–19^ Investigations into the deformation behavior of such simplified membrane and vesicle models provide valuable insights into the mechanical origins and intermolecular interactions that regulate membrane deformation processes essential for biological functions such as protein sorting and signal transduction. Furthermore, the opening and closing of pores in vesicles, reported in earlier studies, suggest the potential for encapsulating various substances, enabling vesicles to act as microreactors that mimic the behavior of living cells.

Similar to the microstructures found in LyLCs,^20–22^ those observed in ThLCs are also described in terms of a competition between interfacial and elastic energies.^4,5,23–27^ Peddireddy *et al.* reported that cooling induces a transition from a droplet to a fiber structure, followed by a reversible return to the initial droplet structure upon heating.^4^ Thus, ThLCs have been reported to undergo shape changes solely in response to temperature variations. These findings suggest that ThLC-based systems may not only function as soft photonic elements but also serve as physical models for studying active lipid regulation and cytoskeletal dynamics in biological systems.

From a numerical standpoint, morphological deformation behaviors, including disc-like and umbrella-like structures, have been reproduced using rigid rod-like molecules that exhibit properties similar to those of ThLC.^28–32^ However, experimentally, the deformation behaviors of ThLC-based microstructures reported so far remain limited to only a few patterns, and systematic experimental studies are still scarce.^2,4,33,34^

In this study, we found that two independent systems can make LC fiber structures undergo structural transitions into disc-like and umbrella-like structures. The systems consisted of mixtures of ThLC and a small number of additives. Using temperature stimulation, we successfully controlled the transition between umbrella-like and disc-like structures in a reversible manner. Keeping the universal aspect of the shape transition observed in two different systems, we introduced a general, yet straightforward mathematical model based on a free energy. We can show that the essence of the deformation can be explained with the topological defect energy and the elastic energies of the membrane.

## Experimental

### Materials

We purchased 4-cyano-4′-*n*-octyloxybiphenyl (8OCB; Tokyo Chemical Industry Co., Ltd), decanol (1-decanol, Fujifilm Wako Pure Chemical Corporation), 4-[(6-acryloyloxy)hexyloxy]-4′-cyano-biphenyl (A6CB; Tokyo Chemical Industry Co., Ltd), 1-oleoyl-*rac*-glycerol (monoolein; Sigma Aldrich), pyrromethene 580 (PM580; Luxottica Group S.p.A.), and hexadecyl trimethyl-ammonium (CTAB; Tokyo Chemical Industry Co., Ltd) for use without further purification. The masses of the samples were measured using an analytical balance (AUX220, Shimadzu, AB104-S, Mettler Toledo). The 8OCB undergoes an isotropic to nematic transition at *T*_I–N_ = 80 °C and a nematic to smectic-A transition at *T*_N–SmA_ = 67 °C.^35^

### Sample preparation and observation setup

In this study, we observed two different systems: system A uses 8OCB, decanol, and A6CB, and system B uses 8OCB, monoolein, and CTAB.

#### System A

We used a mixture of 2.9 × 10^−4^ mol 8OCB, 0.3 × 10^−4^ mol A6CB, and 3.2 × 10^−4^ mol decanol. The mixture was heated to 70 °C using a hot plate (C-MAG HS4, IKA; or PC-420D, Corning) until it was fully melted. We then placed 10 µL of the mixture on a glass slide which was set on a temperature-controlled microscope stage (T-96-P, Linkam Scientific Instruments Ltd; or LK-600PH, Linkam Scientific Instruments Ltd). The temperature was set to 45 °C and maintained for 2 min. Subsequently, the temperature was decreased at a rate of 3 °C min^−1^ and cooling was stopped at 34 °C. The temperature was maintained at 34 °C for 10 min. Then, the temperature was decreased at a rate of 0.2 °C min^−1^ approximately 5 min for the growth of LC fiber structures.

After the above procedure, to observe the elongation and shrinkage of the LC fiber structures, we then applied a cyclic temperature protocol. The protocol consists of a programmed cooling rate of 0.2 °C min^−1^ for approximately 2 s, holding the temperature constant for approximately 2 s, and repeating this cycle. When the nucleation of the disc-like structure appeared, the temperature was kept constant to induce the transitions from the LC fiber structure to the umbrella-like structure.

Various heating and cooling processes described in the Results section were applied after the microstructure had achieved an umbrella-like structure to replicate the microstructures (umbrella-like and disc-like structures) described in the main text.

The structural transitions were observed using a polarizing optical microscope (POM; Lv100NPOL, Nikon; or BX51, Olympus) equipped with digital cameras (LUMIX DC-G9, Panasonic; or Digital Sight 1000, Nikon).

#### System B

We used a liquid-crystal (LC) mixture and an aqueous phase. The LC mixture consisted of 8OCB with 2 wt% monoolein, and the aqueous phase was water containing 0.5 mmol L^−1^ CTAB. The LC mixture was heated to above 80 °C on a hot plate (C-MAG HS4, IKA; or PC-420D, Corning) until it was fully melted. In this LC mixture, complete dissolution can be achieved at temperatures above 80 °C. Then, 60 µL of the aqueous phase was placed on a glass substrate at room temperature, followed by deposition of 1 µL of the LC mixture on the aqueous droplet. A cover glass was placed on top to form a sample cell, and the edges were sealed with epoxy glue.

For the observation using a spinning-disk confocal microscope (a Nikon Eclipse Ti microscope with CrestOptics S.p.A X-LIGHT V2/V2 LFOV), we used the essentially same sample with 0.2 wt% of the fluorescent dye PM580.

Since the cell was prepared at room temperature (21 °C), the LC mixture, which had been at 80 °C, was cooled during the preparation process. As a result, when the cell was completed and the LC microstructures were observed, the sample temperature was 36 °C.

For the observations using a polarizing microscope (BX51, Olympus), images were recorded with a digital camera (Digital Sight 1000, Nikon). The sealed cell was placed on a temperature-controlled stage (LK-600PH, Linkam Scientific Instruments Ltd) and held at 80 °C for approximately 2 min. The temperature was then decreased from 80 °C to 30 °C at a cooling rate of 20 °C min^−1^ to generate the LC fiber structures. The definitions of the setting rate and effective rate are provided later in this section. After allowing the LC fiber structures to equilibrate at 30 °C, a structural transition from the fiber to an elliptic disc-like structure was induced, and subsequent heating promoted a transition from the elliptic to a circular disc-like structure. After the microstructure transformed from the fiber structure into the umbrella-like structure, we applied temperature increases and decreases to induce reversible structural transitions between the umbrella-like and disc-like structures.

In the present study, morphological changes at constant temperature following the temperature protocol were performed using a temperature control stage (T-96-P, Linkam Scientific Instruments Ltd). For experiments investigating temperature-induced morphological changes of the microstructures during cooling and holding or heating, a temperature control system (LK-600PH, Linkam Scientific Instruments Ltd) was used. In this Linkam system, relatively large temperature fluctuations are observed after the temperature is set. The temperature sensor was mounted on the sample stage containing the heating element. The heating and cooling rates were programmed in the temperature controller, and the temperature sweeps were carried out automatically. During the first few seconds after the temperature is set, the actual temperature change differs from the value programmed in the temperature controller. Therefore, when using the LK-600PH temperature control system, the temperature values displayed on the controller were extracted using an optical character recognition (OCR) method. Based on the time-resolved temperature values obtained from the display, we made the temperature profile. Therefore, for experiments performed using the LK-600PH temperature control system, both the programmed temperature rate and the actual temperature profile are reported.

The LC microstructures in systems A and B were analyzed using Image J software (Wayne Rasband, NIH).^36^

## Results and discussion

### Transitions of the microstructure in system A

**Fig. 1:**
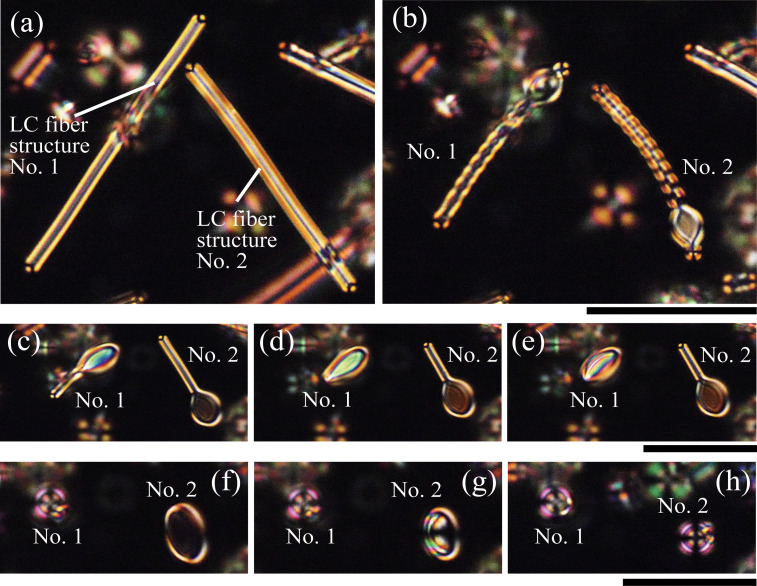
Observation of the structural transition process of LC fiber structures produced using system A. These images were taken under a POM. The temperature was kept at around 33 °C for (a) 0 s, (b) 10 s, (c) 61 s, (d) 77 s, (e) 78 s, (f) 88 s, (g) 91 s, and (h) 117 s. (a) and (b), (c)–(e), and (f)–(h) each use the same scalebar. Each scale bar represents 60 µm.

System A uses the mixture of decanol/8OCB/A6CB. As shown in Fig. 1, we observed microstructures in a continuous phase. Based on the phase behavior of the current system,^25^ we can assume that the microstructure is in the smectic-A (SmA) phase, while the surrounding continuous phase is in the isotropic phase under our experimental conditions. For detailed temperature–composition phase diagrams, please refer to the study by D. Sato *et al.*^25^

In our system A, we initially observed a LC structure, as shown in Fig. 1(a), before the repetitive temperature changes. By applying a cyclic temperature protocol as described in the preceding section (using a T-96-P temperature control system), we observed the elongation of the LC fiber structure upon cooling and shrinkage at the constant temperature. After several repetitions of this process, the LC fiber structure showed structural changes rather than simple length shrinkage at the constant temperature. Once we observed the structural change indicated by the defect, as shown in Fig. 1(b), the temperature was kept constant.

**Fig. 2:**
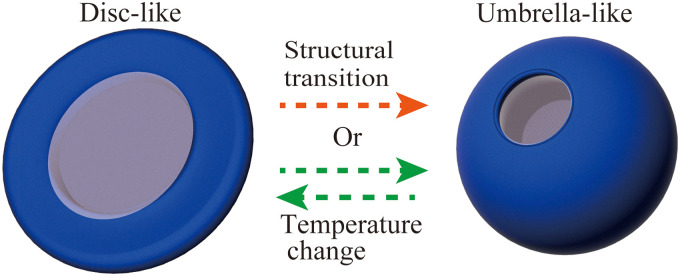
Schematic illustration of the deformation dynamics of LC microstructures. The structural transition process (Fig. 1(f)–(h), LC structure no. 2) at constant temperature after the initial cyclic temperature change of the LC fiber structure is indicated by the orange arrow. The shape transition process at constant temperature which will be shown in Fig. 4 (Fig. 4(e) and (f)) is also indicated by orange arrows. The reversible structure due to the temperature change is indicated by the green arrow which will be shown in Fig. 3 and 5.

Fig. 1 shows the change in the morphologies of the LC microstructure: from fiber to umbrella-like *via* the disc-like structure. Both the samples, no. 1 in Fig. 1(d) and no. 2 in Fig. 1(f), first converged into a disc-like structure. They were eventually converged into a more spherical structure, as shown in Fig. 1(h). The spherical structure was later confirmed to be umbellar-like, as schematically shown in Fig. 2. When the temperature protocol was applied using the LK-600PH temperature control system, we similarly observed a structural transition from fiber through the disc-like structure and finally to the umbrella-like structure (Fig. S1).

Here, we describe the typical transition sequence using sample no. 2 as an example. During the transition process from fiber to the disc-like structure, the disc-like structure appeared from a portion of the LC fiber structure (Fig. 1(b)–(f)). Interestingly, the fiber part was spontaneously twisted in the early stage of deformation (Fig. 1(b)). The fiber part gradually shortened, and the twist loosened, eventually forming a disc-like structure (Fig. 1(f)). However, the structure was further transitioned to an umbrella-like structure (Fig. 1(h)). The snapshots in Fig. 1(f)–(h) show that the disc first adopted a concave structure and then appeared to converge into a spherical form. These observations indicate that the final structure is not a droplet but an umbrella-like membrane: a hollow spherical structure with a pore that connects the interior to the exterior. We sometimes observed that the twist appeared at the rim of the disc-like structure, as shown in Fig. S2(e). In this case, the microstructures did not show the transition to an umbrella-like structure.

In terms of topological charge observed in the microstructure, the initial LC fiber structure had a +1/2 charge at both ends (Fig. 1(a)). In the disc-like structure (Fig. 1(f)), there would be +1 charge on each end and −1/2 charge on the inside; see Fig. S3 for a detailed schematic presentation of topological charges.

**Fig. 3:**
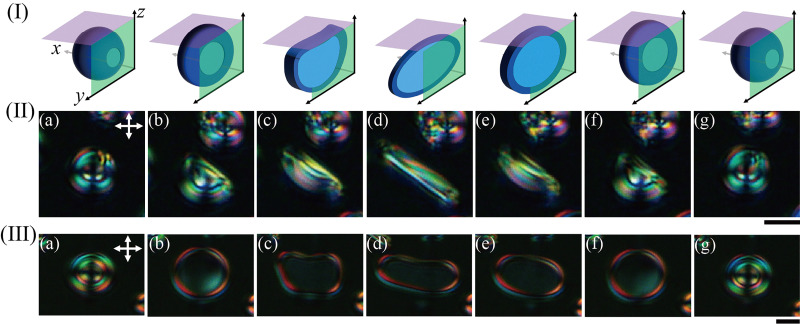
Temperature control of structural transition in LC microstructures produced using system A. These experiments were conducted using a temperature control system (LK-600PH, Linkam Scientific Instruments Ltd). (I) Schematic diagram of deformation. These images illustrate the deformation processes corresponding to each column (a)–(g) in (II) and (III). (II) show the top view of deformation in the *X*–*Y* plane ((I) purple surface), and those in (III) show the side view of deformation in the *Y*–*Z* plane ((I) green surface). II(a)–II(c) and III(a)–III(c) show the cooling process (programmed cooling rate of 0.2 °C min^−1^), and the others (II(d)–II(g) and III(d)–III(g)) show the holding process. The figures were taken by POM. II(a) 0 s, II(b) 3.2 s, II(c) 4.3 s, II(d) 7.5 s, II(e) 8.5 s, II(f) 9.4 s, and II(g) 12.9 s. III(a) 0 s, III(b) 2.8 s, III(c) 4.0 s, III(d) 7.4 s, III(e) 9.0 s, III(f) 9.8 s, and III(g) 13.8 s. The scale bars represent 10 µm.

Fig. 3 shows the repetitive structural changes between umbrella-like and disc-like structures during the cooling process (a)–(d) and holding process (d)–(g). In Fig. 3, we applied a programmed cooling rate of 0.2 °C min^−1^ for 5 s and then kept the system at a constant temperature. For the detailed temperature profile, please refer to the Fig. S4(a). Fig. 3(II) and (III) also present the deformation processes of two samples observed in the same cell. Fig. 3(II) and (III) correspond to the cross-section of the purple (*xy*) and green (*yz*) plane in the schematic diagram of Fig. 3(I). Therefore, the planar-looking shape observed during the deformation process, as shown in Fig. 3(II(d)), would be consistent with the disc-like structure shown in Fig. 3(III(d)). As shown in Fig. 3(II) and (III), we confirmed that after the structure was transformed from an umbrella- to a disc-like structure by decreasing the temperature, it returned to its initial umbrella-like structure by holding the system (Fig. S5). In other words, the umbrella-like structure is suggested to be thermodynamically more stable than the disc-like structure.

To observe the reversible transition between the umbrella-like and disc-like structures, as shown in Fig. 3, it is first necessary to generate the umbrella-like structures through the morphological transition from a LC fiber structure, as depicted in Fig. 1. In some instances, cooling the umbrella-like structure caused it to pass through the disc-like structure and eventually transform back into the fiber. This behavior suggests that reversible structural transitions can also occur between the umbrella-like and the fiber structures.

### Transitions of the microstructure in system B

**Fig. 4:**
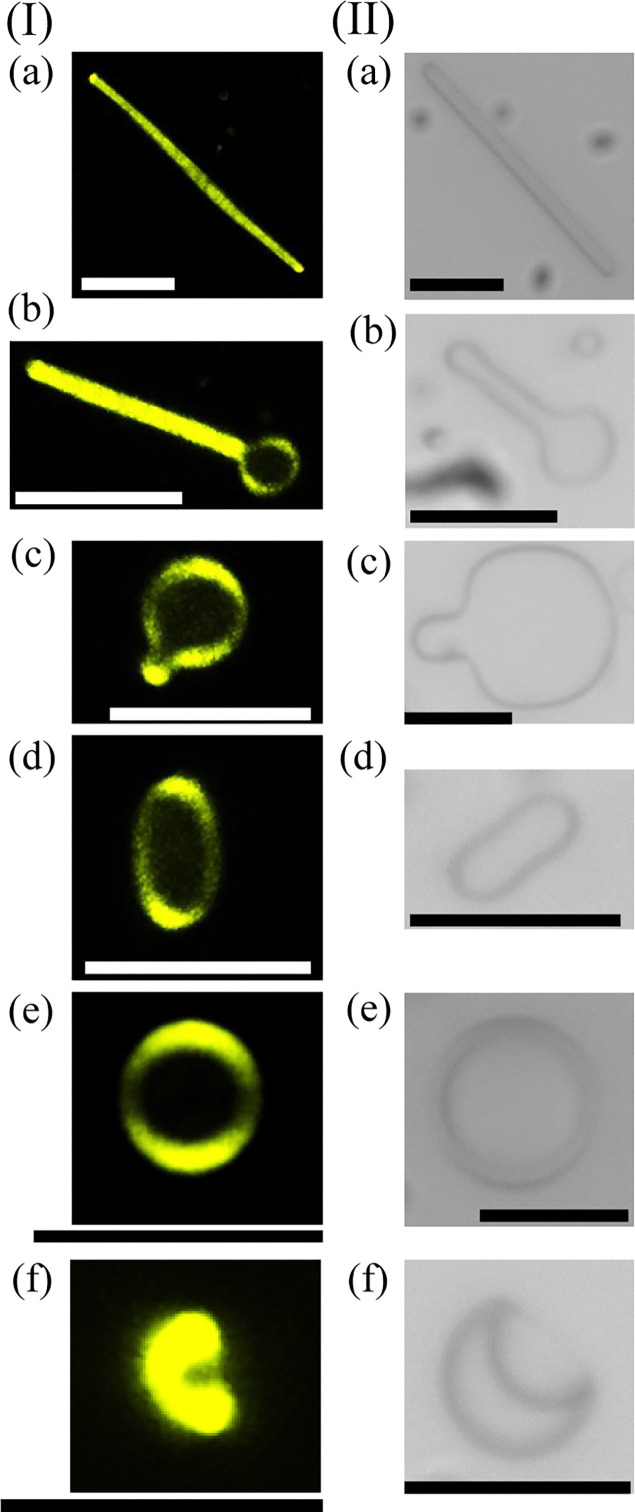
Observation of the structural transition process from the fiber to umbrella-like structure using system B. (I) Observation of transitions in LC microstructures using a high-speed confocal microscope. The temperature was kept at 36 °C. (II) Time series of transition of LC microstructures observed using a phase contrast microscope. The same Roman letters represent the corresponding structure. The scale bars represent 10 µm.

Fig. 4 shows the structural transition of the LC fiber structures generated in system B at a constant temperature of approximately 36 °C using a fluorescence confocal image. As in system B, the LC microstructures observed in this experiment are considered to be in the SmA phase.^26^ Detailed DSC measurements and corresponding POM images for the 8OCB/monoolein mixtures can be found in Fig. S6.

We observed that the structural transitions in Fig. 4 progressed from a fiber to the umbrella-like structure (Fig. 4(a)–(f)) through the disc-like structure (Fig. 4(d) and (e)), as observed in system A. We note that twisting deformation was not observed in the LC fiber structures, different from those in system A (Fig. 1(b)).

**Fig. 5:**
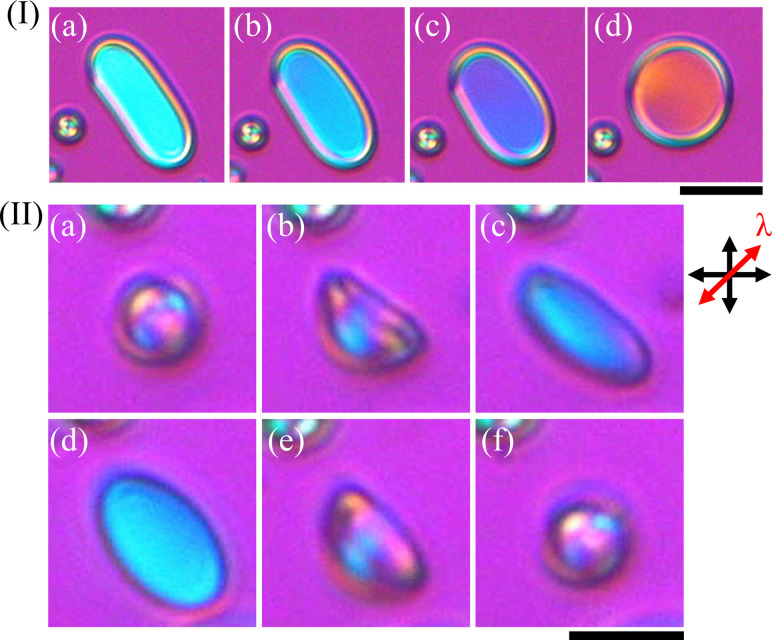
Transformation of the umbrella-like to disc-like structure and change in interference color *via* temperature change using system B. (I) and (II) were observed with the POM + λ plate. I(a)–(d) show the heating process and the programmed heating rate was 5.0 °C min^−1^. I(a) 0 s (27.3 °C), I(b) 4.1 s (27.6 °C), I(c) 7.6 s (27.8 °C), and I(d) 12.7 s (28.1 °C). The temperature values were described using the actual heating rate estimated by the OCR method. II(a)–(c) are in the cooling process (programmed cooling rate of 30 °C min^−1^) and II(d)–(f) are in heating process (programmed heating rate of 30 °C min^−1^). II(a) 0 s, II(b) 4.1 s, II(c) 5.2 s, II(d) 16.4 s, II(e) 17.2 s, and II(f) 20.2 s. The scalebars represent (I) 20 µm and (II) 10 µm respectively.

Using Fig. 4(I), 5(I), and Fig. S7, we now examine the area enclosed by the rim. The areas surrounded by rims look dark (Fig. 4(I(b))–(I(e))). In contrast, in Fig. S7 and Fig. 5(I), the interference color within the region enclosed by the rims appears bright cyan when the microstructure is elliptical (Fig. 5(I(a))) and gradually shifts to orange as the structure approaches circular (Fig. 5(I(d))). For the detailed temperature profile, please refer to Fig. S4(b). This observation indicates that LC molecules are also present in the rim-enclosed region. The fluorescent dye (PM580) used in our experiments is known to exhibit strong affinity for LC molecules,^37^ and thus the fluorescence intensity reflects the local density of LC molecules. Taken together, the results in Fig. 4(I), 5(I), and Fig. S7 make it evident that the rim-enclosed region is not a ‘hole part’ but is instead composed of a thin LC layer. Based on the observations shown in Fig. 5(I) and Fig. S7 the thin LC layer exhibits a uniform interference color, suggesting that the director, *i.e.*, 8OCB LC molecules, is uniformly aligned along the direction perpendicular to the observation direction, *i.e.*, in the focal plane of the microscope.

Fig. 5(II) shows the reversible transition between an umbrella-like and a disc-like structure at the programmed cooling rate of 30 °C min^−1^ and the programmed heating rate of 30 °C, observed under a POM with a λ-plate inserted. For the detailed temperature profile, please refer to Fig. S4(c). During the cooling process from the umbrella-like structure (Fig. 5(II(a))), we found the enlarged pore (Fig. 5(II(b)) and (II(c))) as its shape converged to be a disc-like structure (Fig. 5(II(d))). We should emphasize that this transition was also reversible. The pores were closed (Fig. 5(II(e))), and the shape changed to an umbrella-like structure (Fig. 5(II(f)) when the system was heated. In Fig. 5(II(c)) and (II(d)), the light blue interference color was observed in the disc-like structure. As described in the previous paragraph, this interference color indicates that the central region of the disc-like structure is composed of LC layers. Finally, we mention here that the transition from the disc- to the umbrella-like structure occurred even when the system was maintained at a constant temperature, as observed in system A.

The initial transition of the LC microstructures in systems A and B (Fig. 1 and 4) and the cyclic transition in systems A and B (Fig. 3 and 5(II)) are schematically indicated by orange and green arrows in Fig. 2. In both systems, we observed a characteristic sequence of morphological changes, including the transformation from a fiber to an umbrella-like structure and the reversible transition between umbrella-like and disc-like structures. This similarity suggests that the fundamental mechanism of the shape transitions is governed mainly by generic physical factors, such as energy balance, rather than by the specific chemical components of each system. We also note that the transition from the fiber to the umbrella-like structure occurred less frequently in system B than in system A. This difference can be attributed to the fact that system A is an oil-in-oil system, whereas system B is an oil-in-water system, so that the microstructure in system A experiences lower interfacial tension. Consistent with this interpretation, we observed re-elongation of the fiber structure from the umbrella-like structure more frequently upon cooling in system A.

### Response of LC microstructures to temperature changes

**Fig. 6:**
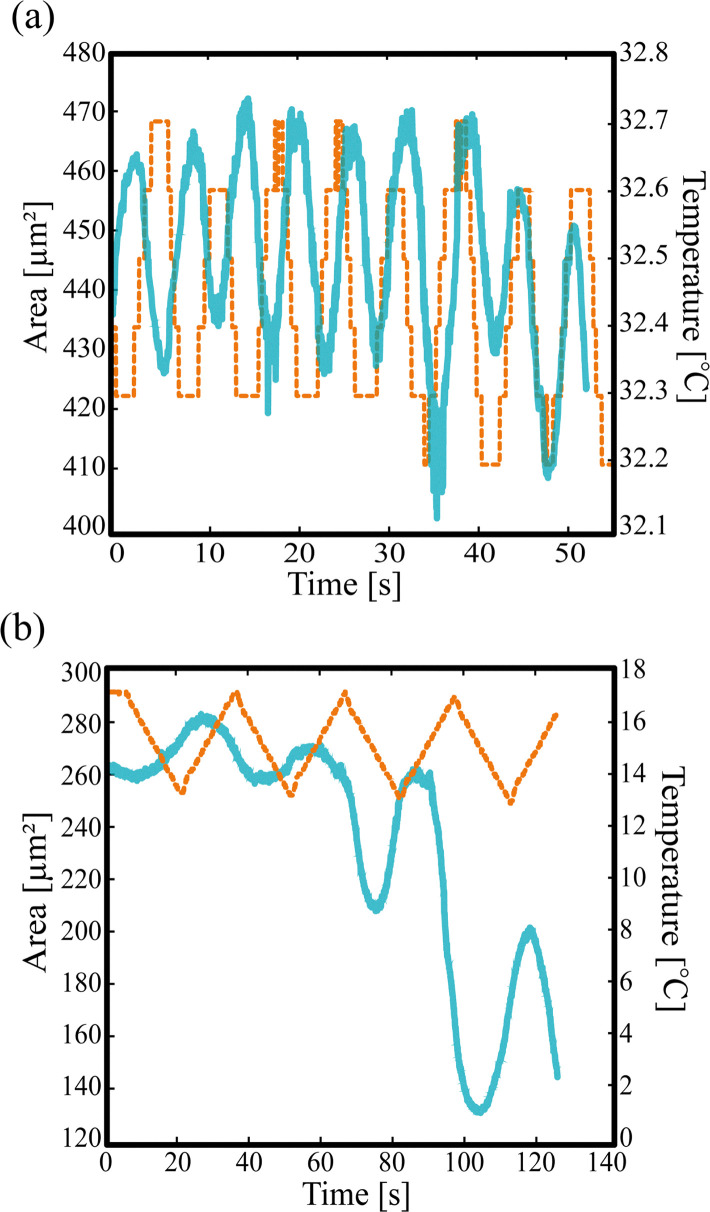
Change in area of LC microstructures with the temperature change. The orange line on the graph represents the temperature change, and the blue line represents the area change. These experiments were conducted using a temperature control system (LK-600PH, Linkam Scientific Instruments Ltd). (a) In case of system A, we repeated cooling and heating for 3 seconds each. The actual cooling and heating rates were both 7.7 °C min^−1^ (programmed cooling and heating rate of 0.2 °C min^−1^). (b) In case of system B, we repeated cooling and heating for 15 seconds each. The actual cooling and heating rates were both 14.5 °C min^−1^ (programmed cooling and heating rate of 16 °C min^−1^).

In what follows, we present an analysis of the apparent area of microstructures accompanied by structural transition. Fig. 6 shows an example of the change in area of LC microstructures in response to temperature stimuli in systems A (Fig. 6(a)) and B (Fig. 6(b)). In both systems, the visually estimated area of the LC microstructures increased during cooling and decreased during heating with a small delay. In system A, the visually estimated area change ranged from 420 to 470 µm^2^ within the observation period, at an actual temperature increase/decrease rate of approximately ±7.7 °C min^−1^ estimated using an OCR method (programmed temperature increase/decrease rate: 0.2 °C min^−1^), as shown in Fig. 6(a). During the 1.5 temperature cycles (2.6–11.6 s), the visually estimated area changed by an average of 36 µm^2^, and the visually estimated change per unit temperature-change rate, *χ*_A_, was 4.7. In system B, the visually estimated area change was approximately 140 to 280 µm^2^ within the observation period, at an actual temperature increase/decrease rate of around ±14.5 °C min^−1^ estimated using OCR methods (programmed temperature increase/decrease rate of 16 °C min^−1^), as shown in Fig. 6(b). During the 1.5 temperature cycles (7.8–84.0 s), the visually estimated area changed by an average of 20.2 µm^2^, and the visually estimated change per unit temperature-change rate, *χ*_B_, was 1.4. When the analysis was unified over 1.5 temperature cycles (Fig. 6), we found that *χ*_A_ > *χ*_B_, indicating that the spatial modulation in the amplitude of the area in system A was larger than that in system B in response to cooling and heating. Furthermore, in system B, the area gradually decreased as the temperature changed repeatedly. We observed that the samples eventually converted to an umbrella-like shape after 3 cycles. Fig. 6 shows that both systems A and B have a disc-like structure up to a certain temperature cycle, but in system B, the shape eventually converges to an umbrella-like structure as the temperature cycle is repeated. In contrast, in system A, the area changes with temperature while maintaining a disc-like structure within the observation range.

Such a difference between systems A and B could be attributed to the following two reasons. The first reason is the lower interfacial tension between the microstructure and the surrounding continuous phase in system A. Indeed, system A has an organic LC droplet within the organic continuous phase, while the organic LC phase was dispersed in the aqueous phase in system B. Therefore, system A would have a lower interfacial tension than system B, making it easier to deform. In addition, we would like to suggest the other possible reason for the difference between systems A and B, the adsorption rate of the LC molecule onto the microstructure. However, before we discuss this possibility, we start from the discussion on the free-energy based argument on the stability of the microstructure at constant temperature.

### Free-energy-based stability analysis of microstructures

**Fig. 7:**
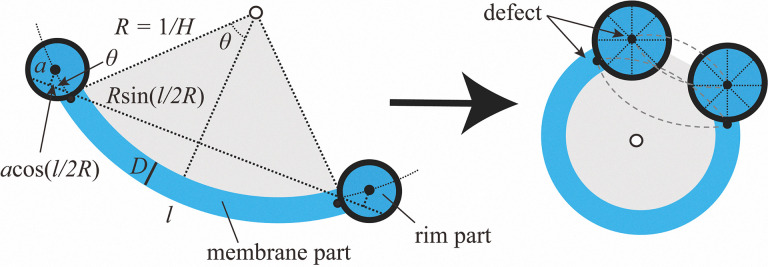
Schematic drawings used in mathematical calculations. The figures show two-dimensional cross sections.

To account for the stability of disc- and umbrella-like structures, we present a simple mathematical model (Fig. 7) within a free-energy framework. We adopt the Helfrich model,^38–40^ which is widely used to describe the deformation of LyLC vesicles^41–44^ and of membranes made of rod-like molecules similar to ThLCs.^24–28^ The essence of our model is illustrated in Fig. 7. The microstructure, either the disc-like or umbrella-like structure, is assumed to have a membrane and a rim part as in Fig. S8. The total free energy, *F*_total_, of this system is written as

1 *F*_total_ = *F*_elastic_ + *F*_defect_

here, *F*_elastic_ is the bending energy of the membrane part and *F*_defect_ is the energy for the defect lines at the middle of the rim and the junction between the rim and the membrane parts (Fig. 7 and Fig. S3). The interfacial energy of the membrane is given by *F*_interface_ = *γA*, where *γ* and *A* are the interfacial tension and the area for the membrane part, respectively. In the following, we assume that *A* is constant and therefore neglect *F*_interface_.

*F*_elastic_ depends on the local curvature of the membrane, described by the mean and the Gauss curvatures, *H* and *K*_G_, as2
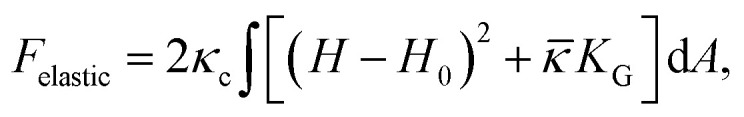
where *κ*_c_, *κ̄*, and *H*_0_ are the bending rigidity, Gaussian modulus, and spontaneous curvature of the membrane.

Owing to two defect lines at the middle of the rim and the junction between the rim and membrane part (Fig. 7 and Fig. S3), they have additional energy that is proportional to the total length of the defect line, *L*. Then,

3 *F*_defect_ = *εKL*.

here, *K* is the elastic constant of LC molecules (splay and bend). *ε* is the geometrical factor given by *ε* = log(*a*/*r*_c_),^3,45,46^ where *a* is the radius of the rim and *r*_c_ is the cut-off radius of the defect core, which is conventionally expressed in the range of 1–10 nm.^46,47^ With this notation, *εK* represents the defect energy observed in the rim per unit length.

From the symmetry consideration, we assume the two main curvatures to be the same, and then we have *H* = 1/*R*. We further assume that the LC membrane prefers zero curvature, such that *H*_0_ = 0. Furthermore, we assume that the LC phase constituting the membrane part was in the SmA phase, and the layer spacing of the SmA layers was taken to remain constant even under the displacement of the membrane. Under this assumption, the bending rigidity of the layers can be considered equivalent to the splay elastic constant of the LC molecules.^48^ Based on the values of the splay elastic constant and the sum of the splay and bend constants, we estimated correction coefficient *β*.^3,49^ Therefore, following Helfrich,^50^ we can set *κ*_c_ ∼ *βKD*. Thus, we obtain4
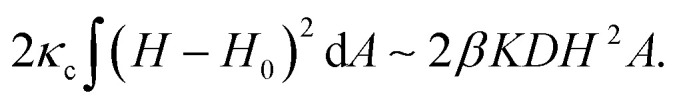


The surface integral of Gauss modulus *K*_G_ on a closed surface is a constant as long as the topology does not change according to the Gauss–Bonnet theorem.^51^ Therefore, in this model, the surface integral term of *K*_G_ in eqn (2) is neglected.

Based on the geometric considerations, *L*(*H*) is expressed as5
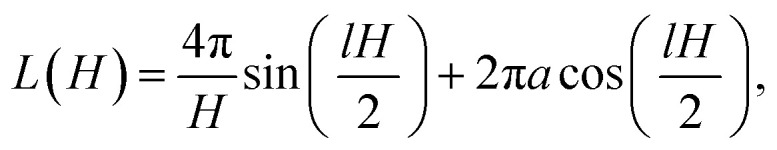
where *l* is the central arc length of the membrane part (Fig. 7).

Using this simplification, we obtained a simplified expression for the free energy of the microstructure as

6 Δ*F* = *F*(*H*) − *F*(*H* = 0) = 2*βKDH*^2^*A* + *εK*{*L*(*H*) − 2π(*l* + *a*)}.

Based on the experimental observations, we find typical values of *l* = 8.6 × 10^−6^ m, *a* = 7.0 × 10^−7^ m, and *A* = 6.45 × 10^−10^ m^2^. In addition, following previous studies, we set the constant values *ε* = 1, *K* = 3.5 × 10^−11^ N and *β* = 0.3.^3,49^

**Fig. 8:**
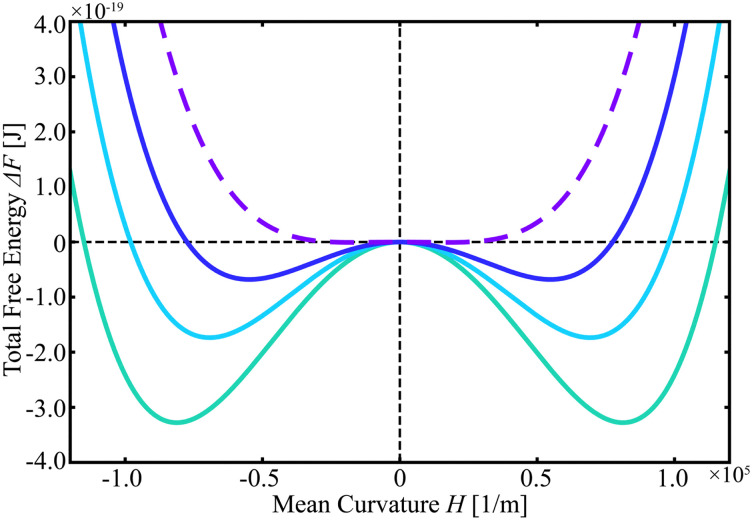
Graph of Δ*F* against the curvature obtained from the mathematical calculation. The thickness *D* was varied as 5.28 (green), 5.3 (cyan), 5.32 (blue) and 5.35 (purple) × 10^−7^ m. The purple dotted line shows when the *D* is 5.35 × 10^−7^ m, where the disc-like structure (with *H* = 0) is stable, whereas the other solid lines show the profile where the umbrella-like structure with finite *H* is stable.

Inserting the above experimental values into our model, we obtain the free-energy profile as a function of the mean membrane curvature *H* (Fig. 8). In this calculation, we varied the membrane thickness *D* over the range indicated in Fig. 8. The results suggest that when *D* is larger than the threshold value ∼5.35 × 10^−7^ m, the flat disc-like structure with *H* = 0 is stable, whereas for small *D*, the minimum of the free energy shifts to a finite *H*, corresponding to the umbrella-like structure. We note that the parameter of *D ∼* 5.3 × 10^−7^ m corresponds to the 260 layers of the smectic A phase.^52^ Vertical observations of the disc-like structure using a confocal microscope revealed that the central disc region appeared completely dark (Fig. 4(I(e))), implying that the membrane in the central part is extremely thin, either thinner than the *z*-slice resolution or containing only a very small amount of fluorescent molecules. Furthermore, Fig. S8 shows that *D* is smaller than *a*. These findings are consistent with the estimated value of *D.*

Within this free-energy picture, the transition from the disc- to the umbrella-like structure can be interpreted as being driven by a reduction in the membrane thickness *D* caused by the desorption of LC molecules from the membrane. Indeed, we observed that cooling the system induces a morphological transition from the umbrella- to the disc-like structure. Our previous study showed that the cooling promotes the adsorption of LC molecules onto the microstructure.^3^ In this case, an increase in *D* leads to the transition from the umbrella- to the disc-like structure. The change in free-energy profile illustrated in Fig. 8 is induced by the variation of *D* on the order of 0.07 × 10^−7^ m corresponding to only about 3 layers,^52^ yet this is sufficient to switch the preferred morphology. In this context, the sequential transition from the disc- to umbrella-like structure suggests that, on a long timescale, the microstructure may eventually “collapse” as the membrane thickness *D* continues to decrease.

The strong dependence of the microstructure on *D* also provides a possible explanation for the different temperature responses between systems A and B, as shown in Fig. 6. In system A, the concentration of LC molecules in the continuous phase is higher because there is no aqueous phase, resulting in a larger reservoir of LC molecules around the microstructure. This difference results in faster absorption–desorption kinetics of LC molecules at the membrane. Consequently, *D* can change more rapidly in system A, which, in turn, results in a faster response of microstructural transition in system A.

## Conclusions

In this study, we observed that LC fiber structures made of 8OCB fabricated by two different systems induce structural transition *via* the change in temperature. We used two different systems to obtain the umbrella-like structure through structural transitions from fiber structures. Despite the differences between the systems, the LC fiber structures underwent broadly similar transition processes and eventually converged to an umbrella-like structure. In system A, a twist was observed in the fiber part during the structural transition from the fiber to the umbrella-like structure. Future investigation of the molecular orientation within the fiber structure and the twisted region would provide deeper insight into the detailed mechanism of the deformation toward the umbrella-like structure. In the present experiments, the structural transition from the fiber to the umbrella-like structure was induced by applying a temperature protocol consisting of cycles of cooling and a constant-temperature (heating) to the fiber in advance. It would be interesting to investigate whether a similar structural transition can also be observed when temperature change rates slower than the programmed cooling rate of 0.2 °C min^−1^ are applied. Furthermore, in both systems, applying thermal stimuli, cooling, heating, or maintaining a constant temperature induced reversible transitions between the umbrella- and disc-like structures. As a subject for future work, it will be interesting to investigate whether the morphological transition from the umbrella-like structure to the disc-like structure can occur at cooling rates slower than those used in the present experiments. It would also be interesting to determine whether there exists a threshold cooling rate below which the umbrella-like structure can be maintained during cooling. In addition, confocal-microscopy observations allowed us to capture the structural transition from the fiber to the umbrella-like structure and to visualize the detailed three-dimensional structure of the umbrella-like structure. Pratibha and Madhusudana reported a similar structural transition: in their study, which was conducted using 8OCB doped with oil, they observed that an LC fiber structure extended by cooling subsequently transformed into an umbrella-like structure when kept at a constant temperature.^3^ However, their work remained limited to reporting the phenomenon, without further discussion or analysis. Peddireddy *et al.* also reported similar deformation behavior, but they did not examine the formation process of the initial umbrella-like structure or investigate the detailed features of the membrane region.^4^ The proposed free energy model in this paper reproduced the experimental behavior, even though it considered only two energetic contributions: the elastic energy of the membrane and the topological defect energy. This simplicity is a key feature of the model, yet it is sufficient to capture the essential characteristics of the observed structural transitions.

In our system, we observed cyclic structural transformations between microstructures formed by ThLCs. Unlike well-known LyLCs, systems based on lipids and surfactants, these microstructures do not exhibit the well-defined bilayer architecture that is typically associated with cellular membranes. In cells, lipid bilayer membranes are ubiquitous and define classical membrane-bound organelles such as the nucleus, mitochondria, and chloroplasts, as well as the cell boundary itself. More recently, however, a different class of organelles, often referred to as membranelles or non-membrane-bound organelles,^53–55^ has attracted considerable attention; these structures arise through liquid–liquid phase separation and regulate the accumulation, compartmentalization, and reactions of biological macromolecules. Our smectic ThLC-based microstructures, which are also generated by phase separation and deform under interfacial and elastic stresses, may therefore serve as simple physical models for such membraneless organelles. From this viewpoint, the cyclic deformations observed in our system illustrate a possible route by which phase-separated, membraneless compartments could exhibit active-like morphological dynamics without relying on biological molecular motors.

## Conflicts of interest

There are no conflicts to declare.

## Supplementary Material

SM-022-D6SM00042H-s001

## Data Availability

Supplementary information (SI) is available. See DOI: https://doi.org/10.1039/d6sm00042h. The authors confirm that the data supporting the findings of this study are available within the article.
