# Supplementary material for: Morphological changes in smectic liquid crystal microstructures
Source: Soft Matter. 2026 Mar 17;22(20):3569–78. doi: 10.1039/d6sm00042h (PMC13045658; doi:10.1039/d6sm00042h)
Supplement: SM-022-D6SM00042H-s001 [file SM-022-D6SM00042H-s001.pdf]

Supplementary Information (SI) for Soft Matter.
This journal is © The Royal Society of Chemistry 2026

Support Information

Morphological changes in smectic liquid crystal microstructures

Daichi Sato,[a,b] Yutaka Sumino,[a,b,c,d] Takahiro Yamamoto,[b] Igor Muševič,[e,f] and Yoshiko Takenaka *[a,b]

[a] *Department of Applied Physics, Graduate School of Science, Tokyo University of Science, 6-3-1, Nijuku, Katsusika-ku, Tokyo, 125-8585, Japan.*
[b] *Research Institute for Sustainable Chemistry, National Institute of Advanced Industrial Science and Technology,1-1-1, Higashi, Tsukuba, Ibaraki, 305-8565, Japan.*
[c] *Water Frontier Research Center and Division of Colloid Interface, Research Institute for Science & Technogy, Tokyo of Science, 6-3-1, Nijuku, Katsusika-ku, Tokyo, 125-8585, Japan*
[d] *Faculty of Engineering and Physical Sciences, University of Surrey, Guildford, Surrey GU2 7XH, United Kingdom.*
[e] *Department of Condensed Matter Physics, Jožef Stefan Institute, Jamova cesta 39, SI-1000 Ljubljana, Slovenia.*
[f] *Physics Department, Faculty of Mathematics and Physics, University of Ljubljana, Jadranska 19, SI-1000 Ljubljana, Slovenia.*

Corresponding: takenaka.yoshiko@aist.go.jp

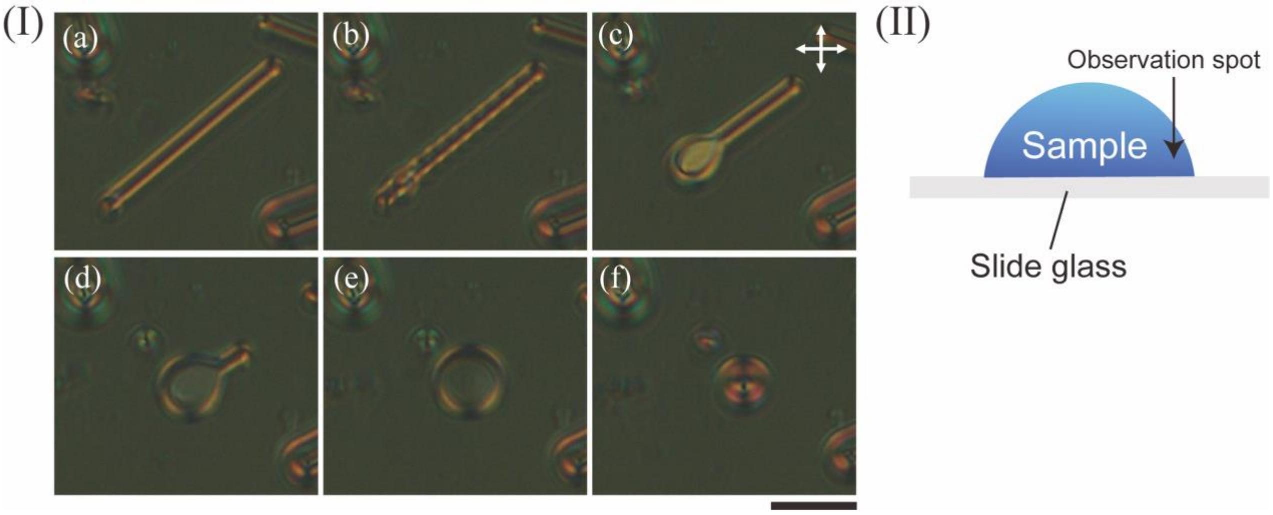

Fig. S1 Observation of the structural transition process of liquid crystal (LC) fiber structures produced using System A using a temperature control system (LK-600PH, Linkam Scientific Instruments Ltd.). (I) POM images under constant temperature at 33.7 °C. (a) 0 s, (b) 2.9 s, (c) 30.0 s, (d) 69.7 s, (e) 83.3 s, and (f) 104.8 s. The scalebar represents 70 µm. (II) Observation set up. LC fibers and the structural transitions were mainly observed near the edge of the sample (indicated observation spot). Because the sample thickness was not uniform along the vertical direction, the continuous phase in (I) did not appear completely dark under POM.

Please do not adjust margins

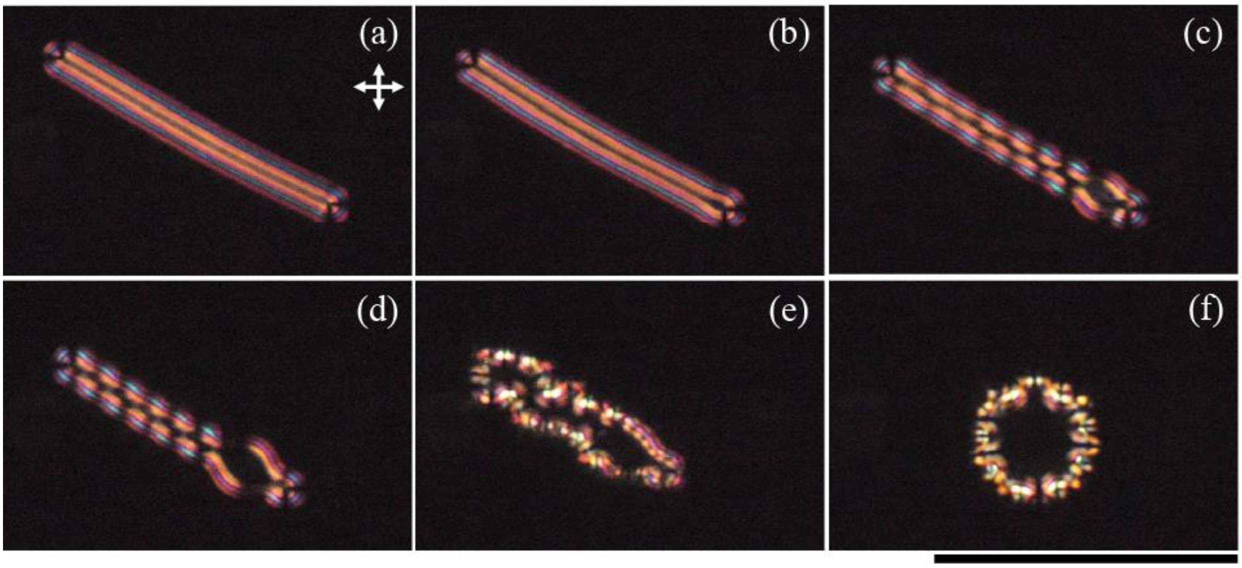

Fig. S2 Aggregation behavior during structural transition of LC fiber structure using System A observed with POM. The experiment was performed using a T-96-P temperature control system (Linkam Scientific Instruments Ltd.). The temperature was not changed during the observations. (a) 0 s, (b) 8 s, (c) 12 s, (d) 18 s, (e) 19 s, and (f) 37 s. The scalebar represents 70 µm.

 This journal is © The Royal Society of Chemistry 20xx

Please do not adjust margins

Please do not adjust margins

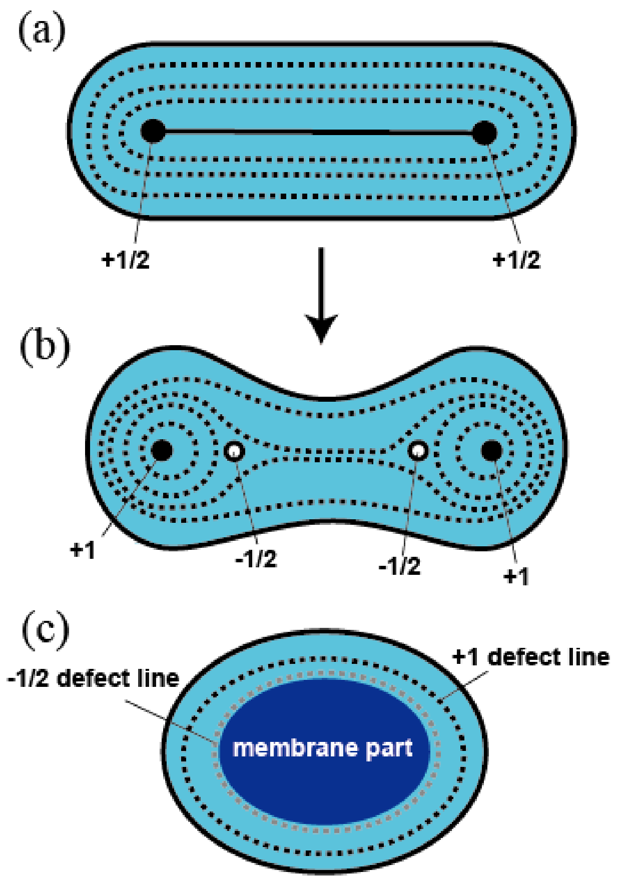

Fig. S3 Changes in topological defects with structural changes. Cross sections of (a) the fiber structure and of (b) the disc-like structure. (c) Top view of the disc-like structure.

Please do not adjust margins

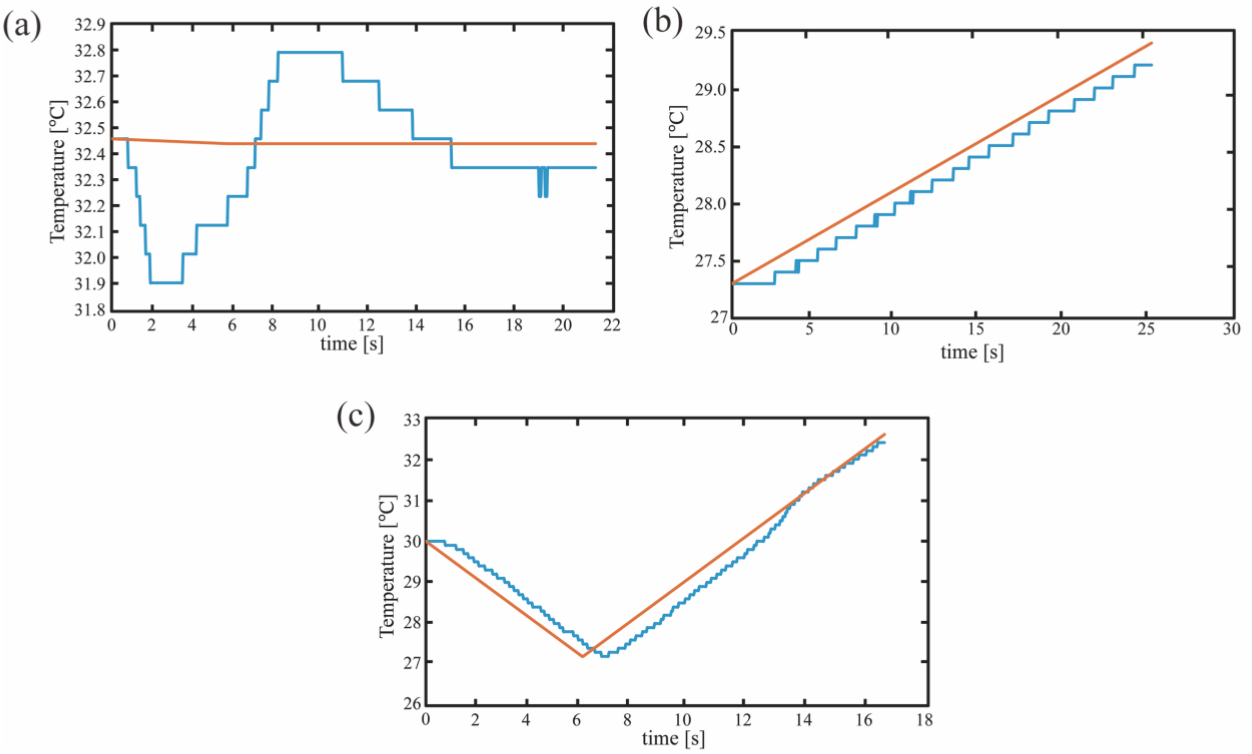

Fig. S4 Temperature profiles corresponding to each experiment. The blue line shows the temperature values at each time obtained using the OCR method, while the orange line represents the ideal temperature profile based on the temperature change rate programmed in the temperature controller. (a) Temperature profile used in the experiment shown in Fig. 3. In Fig. 3, the temperature was cooled for 5 s at a programmed rate of 0.2 °C/min using the temperature controller, after which the controller was set to the temperature holding mode. (b) Temperature profile used in the experiment shown in Fig. 5(I). In Fig. 5(I), the temperature was continuously cooled at a programmed rate of 5 °C/min using the temperature controller. (c) Temperature profile used in the experiment shown in Fig. 5(II). n Fig. 5(II), the temperature was cooled at a programmed rate of 30 °C/min for approximately 5.5 s and then heated at a programmed rate of 30 °C/min using the temperature controller.

This journal is © The Royal Society of Chemistry 20xx

Please do not adjust margins

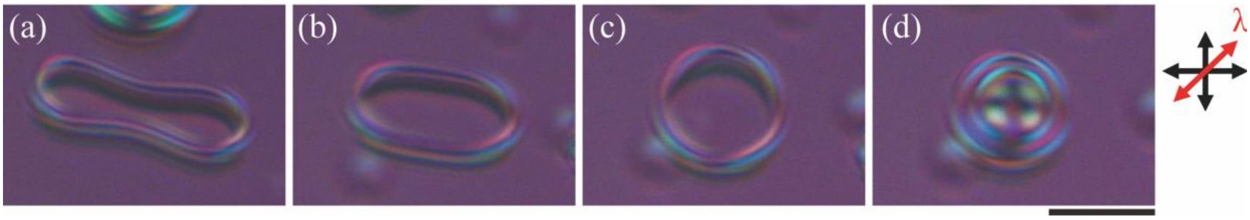

Fig. S5 Observation of the structural transition from a disc-like to an umbrella-like structure under constant temperature using System A. This morphology was observed using POM with λ plate at a constant temperature of 32.4 °C. The experiment was performed using a LK-600 PH temperature control system (Linkam Scientific Instruments Ltd.). The temperature profile obtained using the OCR method shows that temperature fluctuations settle approximately 11 s after the controller is set to the holding mode (data not shown). Image (a) was taken 71.5 s after switching to the holding mode. Although the temperature profile appears to show a slight temperature increase after entering the holding mode, the structural transition from the disc-like structure to the umbrella-like structure was observed under effectively constant temperature conditions. (a) 0 s, (b) 60.0 s, (c) 77.3 s, and (d) 96.7 s. The scalebar represents 20 μm.

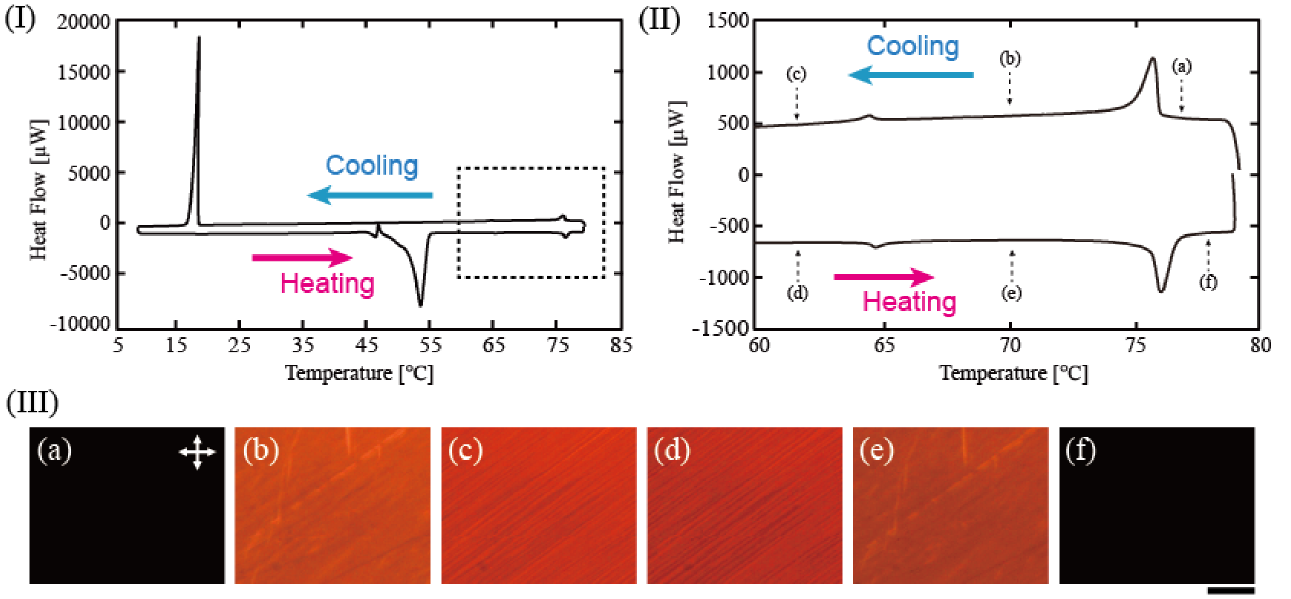

Fig. S6 DSC measurements and corresponding POM images for the 8OCB + monoolein mixture (System B). (I) shows the overall DSC measurement results, and (II) presents an enlarged view of the dotted region indicated in (I). The sample was first held at 80 °C for 10 min, then cooled to 10 °C at a rate of 1 °C/min. After being maintained at 10 °C for 10 min, it was reheated to 80 °C at a heating rate of 1 °C/min. It shows a smectic–nematic transition at 64.5 °C and a nematic–isotropic transition at 75.8 °C. (III) is the POM observations. A cell with a thickness of 2 μm was used. These experiments were conducted using a. LK-600PH temperature control system (Linkam Scientific Instruments Ltd.). The sample was first kept at 80 °C for 10 min, then cooled to 50 °C at a cooling rate of 1 °C/min. After being held at 50 °C for 10 min, it was reheated to 85 °C at a heating rate of 1 °C/min. (a) and (f) are isotropic, (b) and (e) are nematic, and (c) and (d) are smectic A phase. Panels (a)–(f) in (II) correspond to panels (a)–(f) in (III). The scalebar represents 50 μm.

Please do not adjust margins

Please do not adjust margins

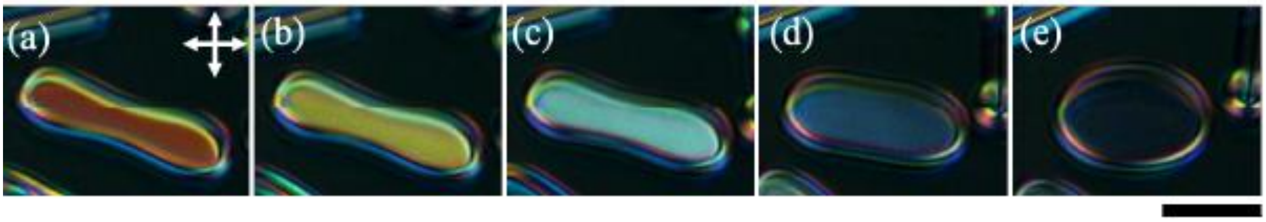

Fig. S7 Time series of color changes in the central part of a disc-like structure. This morphology observed with POM when System A was used. The experiment was performed using a LK-600 PH temperature control system (Linkam Scientific Instruments Ltd.). Temperature was kept at 32.4 ℃. (a) 0 s, (b) 6.2 s, (c) 17.9 s, (d) 45.3 s, and (e) 62.2 s. The scalebar represents 20 µm.

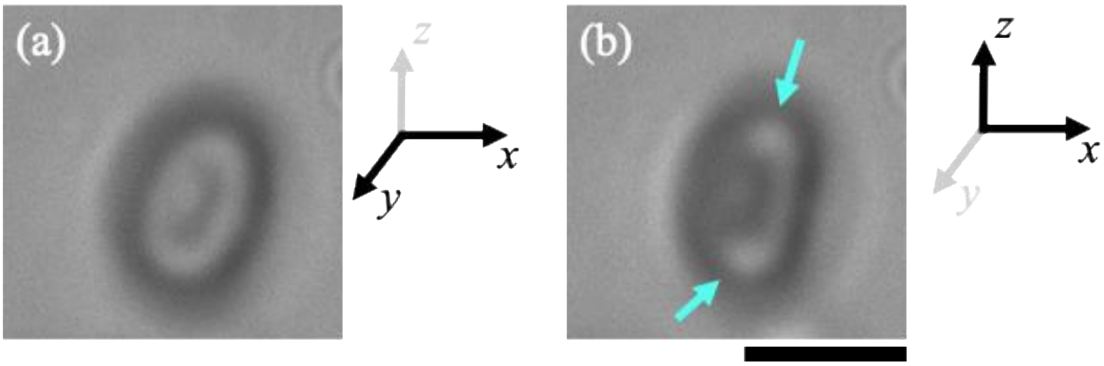

Fig. S8 Multiaxial observation of disc-like structure. These experiments were conducted using a T-96-H temperature control system (Linkam Scientific Instruments Ltd.). (a) Disc-like structure observed in the X - Y plane. (b) Disc-like structure observed in the X - Z plane. The blue arrows in the image (b) indicate the rim at the edge of the disc-like structure in 2D. Both (a) and (b) are observed in bright field. The scalebar represents 50 µm.

This journal is © The Royal Society of Chemistry 20xx

Please do not adjust margins
